# Geographic concordance of cataract and glaucoma burden and associated country-level exposures across 204 countries and territories

**DOI:** 10.3389/fpubh.2026.1901226

**Published:** 2026-10-28

**Authors:** Qiao Chen, Xueying Yang, Liang Li, Changzheng Chen

**Affiliations:** 1 Department of Ophthalmology, Jingzhou Hospital Affiliated to Yangtze University, Jingzhou, Hubei, China; 2 Eye Center, Renmin Hospital of Wuhan University, Wuhan, Hubei, China

**Keywords:** cataract, country-level exposures, geographic concordance, glaucoma, Global Burden of Disease, spatial epidemiology, vision loss

## Abstract

**Background:**

Cataract and glaucoma are major causes of vision loss, but their shared geographic burden and potentially modifiable population-level exposures remain incompletely characterized.

**Methods:**

This cross-sectional study included adults aged 55 years and older across 204 countries and territories, using data from the Global Burden of Disease Study (GBD). For both outcomes, national prevalence was age-standardized using the 2021 global population aged 55 years and older as the reference. Based on quartiles of national age-standardized prevalence for both outcomes, we classified the 204 countries and territories into three geographic concordance categories: Concordant, Cataract-dominant, and Glaucoma-dominant. For 2021, we modeled each of 26 literature-informed candidate summary exposure values (SEVs) separately against log age-standardized prevalence, adjusting for the Socio-demographic Index (SDI) with a three-degree-of-freedom natural spline. Benjamini–Hochberg (BH) correction was applied within each outcome, with additional regional and spatial models used to assess sensitivity.

**Results:**

In 2021, 89 countries (43.6%) were Concordant, 52 (25.5%) were Cataract-dominant, and 63 (30.9%) were Glaucoma-dominant. Fifteen countries were in the highest quartile for both outcomes, mainly in North Africa, the Middle East, and parts of sub-Saharan Africa. Five exposures—ambient particulate matter pollution, ambient ozone pollution, high body-mass index, low bone mineral density, and high temperature—had positive coefficients with outcome-specific BH q < 0.05 for both outcomes in the primary SDI-adjusted models. After adjustment for GBD super-region, only high temperature remained positive for both outcomes with BH q < 0.05. In both spatial-error models, only ambient particulate matter pollution met this standard.

**Conclusion:**

National cataract- and glaucoma-related vision-loss burdens showed geographic concordance. The exposure findings were country-level associations, and their statistical support varied across regional and spatial models. These geographic patterns may help inform planning for cataract and glaucoma services in settings where both contribute substantially to vision-loss burden. Further individual-level research is needed to clarify the relationships suggested by these country-level findings.

## Introduction

Cataract and glaucoma are leading causes of vision impairment and blindness worldwide. Cataract remains the most common cause of blindness, accounting for approximately 17 million blindness cases and 83.5 million cases of moderate to severe vision impairment in 2020 (1). Glaucoma is the leading cause of irreversible blindness; the Global Burden of Disease Study 2021 (GBD 2021) estimated approximately 7.59 million prevalent glaucoma cases in 2021 (2, 3). With population growth and ageing, the absolute burden of both diseases has continued to rise over the past three decades (2, 4). Although their clinical courses and management differ, they frequently converge in older eye-care populations, where delayed diagnosis or fragmented care can compound visual disability.

In ophthalmic practice, coexisting cataract and glaucoma is a familiar problem in older adults (5–7). The overlap reflects more than age alone. Age-related anatomical and functional changes affect multiple ocular tissues and contribute to the increasing burden of age-related eye diseases (8, 9). Diabetes has been associated with both cataract and glaucoma, while long-term corticosteroid use, myopia, smoking, and ultraviolet exposure have been linked to one or both conditions (3, 10–12). Previous studies have also investigated oxidative stress, metabolic changes, and genetic susceptibility in relation to these conditions (13–15). Yet most epidemiological work has considered cataract and glaucoma separately. Much less is known about whether countries with a high cataract burden also tend to carry a high glaucoma burden, or how country-level exposures relate to these burdens.

Clarifying this overlap is relevant to global eye health because cataract services, glaucoma detection, and primary eye-care programmes often rely on the same workforce and infrastructure. A country-level view can show how the two vision-loss burdens align geographically and help inform eye-care planning. We therefore used GBD 2021 data to examine geographic concordance in cataract- and glaucoma-related vision-loss burdens among adults aged 55 years and older in 204 countries and territories. We also assessed associations with country-level candidate exposures after adjustment for the Socio-demographic Index (SDI) and examined their sensitivity to regional and spatial structure.

## Methods

### Study design and data sources

This ecological study examined geographic concordance in the national burdens of cataract- and glaucoma-related vision loss, as well as associations between country-level exposures and each outcome. The primary analyses used 2021 data for adults aged 55 years and older in 204 countries and territories, while additional descriptive analyses examined national burden and concordance patterns from 2011 to 2021. The country or territory was the unit of analysis. Data were obtained from the Global Burden of Diseases, Injuries, and Risk Factors Study 2021 (GBD 2021) database through the Global Health Data Exchange (GHDx). The primary outcomes were national age-standardized prevalence rates of cataract- and glaucoma-related vision loss among adults aged 55 years and older. The glaucoma-related vision-loss outcome was available only as a pooled estimate; separate national estimates for open-angle and angle-closure glaucoma were not available in the analytic dataset. We also collected data on behavioral, environmental, occupational, and metabolic exposure indicators. These exposure indicators were measured using summary exposure values (SEVs) from the GBD framework. Country-level exposures were represented by 2021 aggregate GBD SEVs for adults aged 55 years and older. We also obtained the 2021 Socio-demographic Index (SDI) from GBD and included it as an adjustment variable in the country-level exposure models. The study followed the Guidelines for Accurate and Transparent Health Estimates Reporting (GATHER) statement. Ethical approval was not required because the research used only public, de-identified, and aggregated data from the GBD 2021 database.

### Age standardization

National prevalence was standardized across nine age groups (55–59, 60–64, 65–69, 70–74, 75–79, 80–84, 85–89, 90–94, and 95 + years). For each country, outcome, and year, age-specific prevalence rates were weighted using the fixed 2021 global age distribution among adults aged 55 years and older. Rates are reported per 100,000 population. The resulting age-standardized estimates were used in the descriptive and association analyses, including the regional and spatial models.

### Concordance classification and temporal description

To describe geographic concordance in national cataract- and glaucoma-related vision-loss burden, we used quartiles of country-level prevalence (16, 17). For each outcome and year, the national age-standardized prevalence rates of the 204 countries and territories were divided into four burden tiers: low (below the 25th percentile), lower-middle (25th–50th percentile), upper-middle (50th–75th percentile), and high (above the 75th percentile). The cutpoints were calculated across all 204 countries and territories with equal country weighting. We then grouped countries by comparing their tier assignments for each disease. Countries where both diseases were in the same tier were categorized as the concordant zone. Countries where the cataract burden tier was higher than the glaucoma tier were categorized as the cataract-dominant zone. Countries where the glaucoma burden tier was higher than the cataract tier were categorized as the glaucoma-dominant zone. We used a bivariate choropleth map to show the geographic distribution of these concordance categories (Figure 1C). We repeated the classification using tertiles and quintiles to assess whether the results depended on the number of burden tiers (Supplementary Table S1).

**Figure 1:**
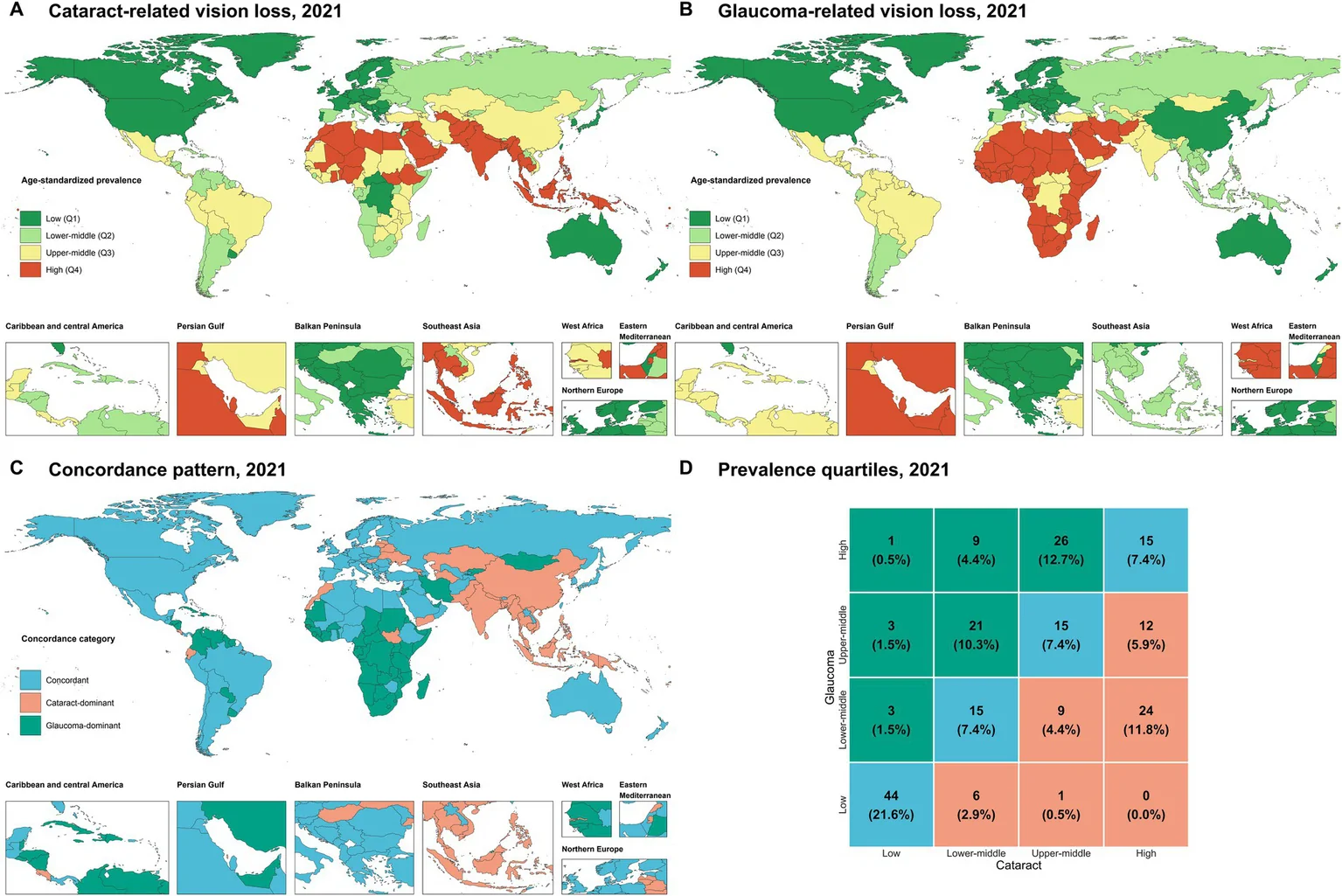
National patterns of cataract- and glaucoma-related vision loss among adults aged 55 years and older, 2021. **(A,B)** Quartiles of directly standardized national prevalence, using the fixed 2021 Global population distribution within ages 55–59 to 95 + years. **(C)** Concordance categories: Concordant, Cataract-dominant and Glaucoma-dominant. The same categorical palette is used in Panels **(C,D)** and Figure 2 and is distinct from the prevalence-tier palette in Panels **(A,B)**. **(D)** Country counts and percentages across the two outcome-specific quartiles. Quartiles are calculated separately for each outcome across all 204 countries and territories. Concordance includes all matching tiers, not only high–high burden. Small island states absent from the polygon layer are represented by points.

For the 2011–2021 analysis, we classified countries using the cutpoints for each outcome in each year (Supplementary Table S2). We mapped the categories in 2011, 2016, and 2021 and summarized their annual counts (Figure 2). We also identified countries that remained in the same category throughout the period and those in the highest quartile for both outcomes in every year (Supplementary Table S3). As a sensitivity analysis, we applied the fixed 2021 cutpoints to all years (Supplementary Table S4). Regional prevalence trajectories were summarized by the median national rate within each of the seven GBD super-regions (Supplementary Figure S1).

**Figure 2:**
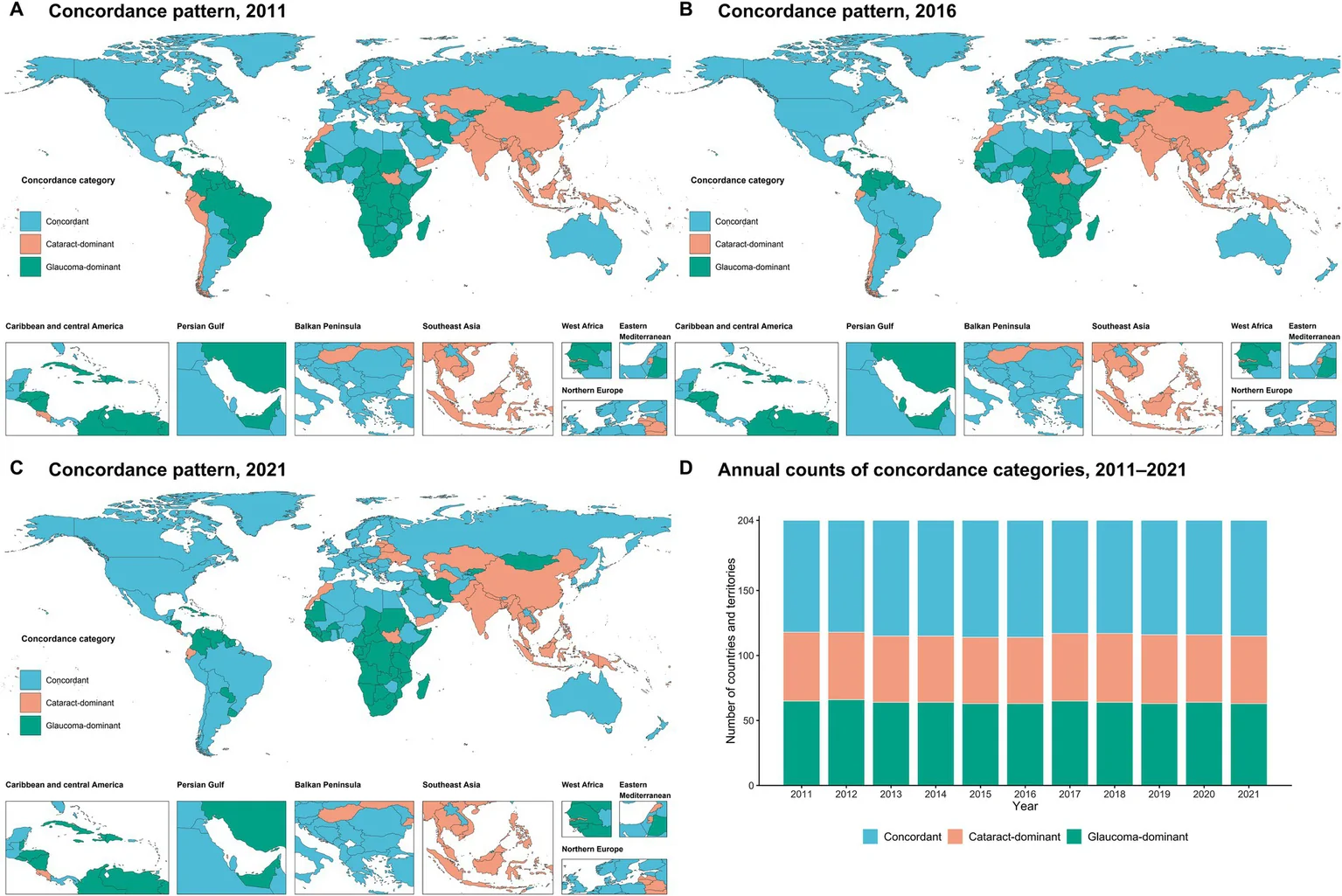
National concordance patterns of cataract- and glaucoma-related vision loss during 2011–2021. **(A–C)** Classifications in 2011, 2016, and 2021. **(D)** Annual counts of the three categories. The same 2021 global population weights are used in every year, while outcome-specific quartiles are recalculated annually across the same 204 countries and territories. Persistence of a concordance category does not imply unchanged disease-specific quartiles, exact ranks, or absolute prevalence. Concordance includes low–low and high–high burden. Fixed-cutpoint results and country stability are reported in Supplementary Tables S1–S4.

### Candidate exposure selection

We considered 58 GBD exposure indicators covering behavioral, environmental, occupational, and metabolic domains. We evaluated all 58 candidate exposures using consistent eligibility criteria based on human epidemiological evidence concerning cataract or glaucoma. We retained an exposure when at least one human epidemiological study used a comparable exposure measure and reported an association with cataract or glaucoma in a direction consistent with the corresponding GBD exposure definition. Mixed findings did not lead to exclusion if these criteria were met.

The literature screen retained 26 of the 58 candidate exposures. Supplementary Table S5 presents the full candidate set, the supporting evidence for retained exposures, and the reasons for non-retention. The same 26 candidates were assessed for both outcomes.

### Primary association models

For each exposure and outcome, we fitted a country-level linear model of log age-standardized prevalence. Each model included one standardized SEV and a three-degree-of-freedom natural spline for SDI, allowing the relationship between SDI and prevalence to be nonlinear. Countries were weighted equally, and each SEV was standardized using the mean and standard deviation across all 204 countries and territories. Exponentiated SEV coefficients are reported as country-level prevalence ratios (PRs) per one standard deviation (SD) increase in SEV.

The 26 candidate exposures were analysed separately for cataract and glaucoma, giving 52 primary models. Each model adjusted for SDI but did not include the other SEVs. We used heteroskedasticity-consistent type 3 (HC3) covariance estimates to calculate pointwise 95% confidence intervals (CIs) and two-sided *p* values, with t-based inference. Benjamini–Hochberg (BH) correction was applied separately across all 26 exposure tests within each outcome. The complete results are presented in Figure 3 and Supplementary Table S6.

**Figure 3:**
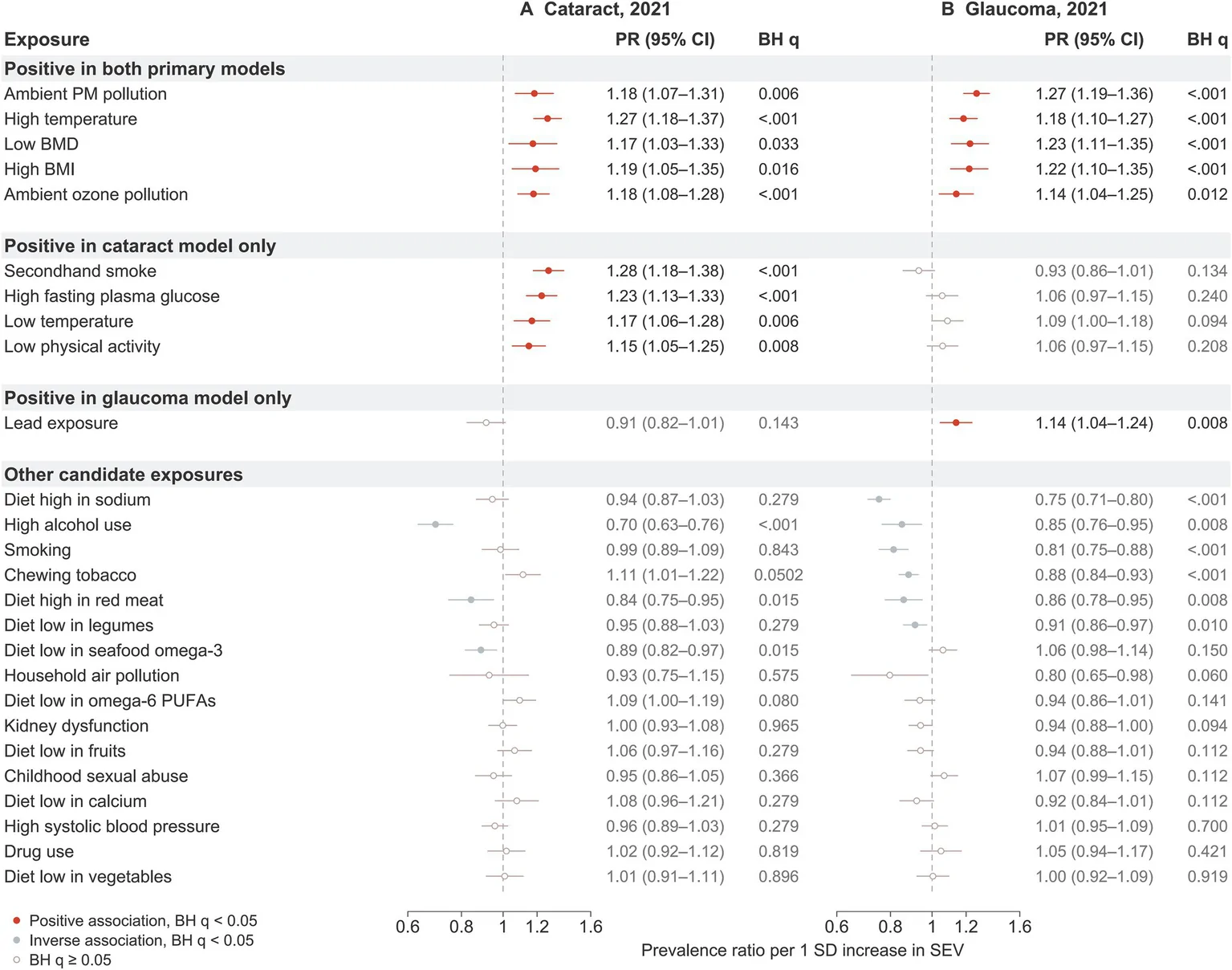
SDI-adjusted associations of 26 literature-informed candidate exposures with cataract- and glaucoma-related vision loss, 2021. **(A)** Cataract; **(B)** glaucoma. All 26 candidates are shown, including inverse and nonsignificant associations. Filled red points indicate positive associations with BH q < 0.05; filled pale blue-grey points indicate inverse associations within “Other candidate exposures” with BH q < 0.05; open points indicate q ≥ 0.05. Points and horizontal intervals show ratios of conditional geometric mean national age-standardized prevalence (PR) per one-SD higher summary exposure value (SEV), with pointwise 95% HC3 confidence intervals, in 204 countries and territories among adults aged 55 years and older. Each model includes one exposure and a three-df natural spline of the Socio-demographic Index (SDI), with equal country weighting. Two-sided t-test *p* values are adjusted separately across the full 26-exposure family for each outcome using the Benjamini–Hochberg procedure. Both panels share exposure order and a logarithmic horizontal scale; the reference line is PR = 1. “Positive in both primary models” requires positive coefficients and outcome-specific BH q < 0.05 in both outcomes. Classification uses unrounded q values. PRs describe country-level ecological associations, not individual risk ratios or causal effects. PM, particulate matter; BMI, body-mass index; BMD, bone mineral density; PUFAs, polyunsaturated fatty acids.

An exposure was described as positive in both primary models when the coefficients were positive and the outcome-specific BH q values were below 0.05 for both outcomes. Positive associations meeting this criterion in only one model were described separately. These categories were descriptive and did not test whether the exposure associations differed between the two outcomes.

### Spatial and regional analyses

We examined spatial patterns in log age-standardized prevalence using univariate Moran’s I for each outcome and directional bivariate Moran statistics relating one outcome in a country to the other outcome in neighbouring countries. The primary spatial weights linked each country to its four nearest neighbours by great-circle distance and were row-standardized; eight-neighbour weights were used for sensitivity analysis. Global significance was assessed using 9,999 permutations (Supplementary Table S7).

To examine the sensitivity of the exposure associations to geographic structure, we refitted all 26 exposure models for both outcomes after adding GBD super-region fixed effects and in spatial-error models with four- or eight-nearest-neighbour weights. Spatial-error models were fitted by maximum likelihood, with covariance obtained from the joint-likelihood Hessian and normal-based inference. The regional models retained a common exposure coefficient while accounting for differences in mean prevalence across super-regions. The spatial-error models retained the primary mean structure, without super-region terms. BH q values were recalculated across all 26 exposures separately for each outcome and model specification. Figure 4 and Table 1 compare the five exposures meeting the primary two-outcome criterion, and Supplementary Table S8 reports the full regional and spatial results. For the five exposures meeting the primary two-outcome criterion, we also refitted the SDI-adjusted models after omitting each GBD super-region in turn (Supplementary Table S9).

**Figure 4:**
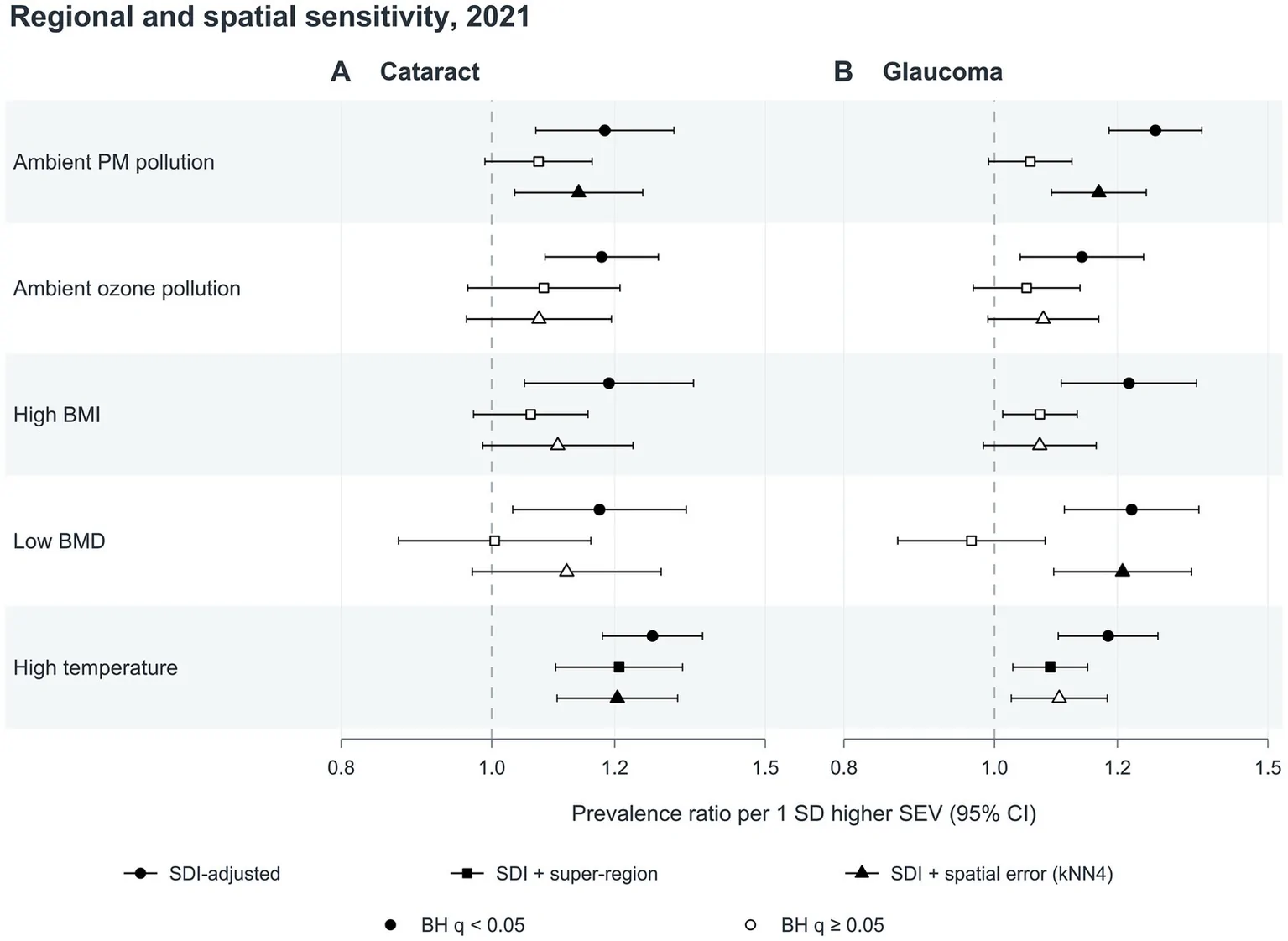
Regional and spatial sensitivity of five primary common-positive associations, 2021. **(A)** Cataract; **(B)** glaucoma. The panels compare three specifications: the primary single-exposure model with a three-df natural spline of SDI; the primary model with additional GBD super-region fixed effects; and a spatial-error model using the primary mean structure with row-normalized four-nearest-neighbour weights (kNN4). Points and bars show PRs per the same full-sample SD of SEV and pointwise 95% confidence intervals on logarithmic horizontal scales. Primary and regional models use HC3 covariance and t-based inference; the spatial-error model uses joint-likelihood-Hessian covariance and normal inference. Marker shapes identify model specifications. Filled points indicate BH q < 0.05 within the complete 26-exposure family for the corresponding outcome and specification; open points indicate q ≥ 0.05. The vertical dashed line marks PR = 1. The confidence intervals are not multiplicity-adjusted. The five displayed exposures were selected using the primary common-positive criterion. Table 1 reports all four specifications for these five exposures, including the eight-nearest-neighbour spatial-error model (kNN8); full results for all 26 exposures appear in Supplementary Table S8.

**Table 1:** Country-level exposure associations across regional and spatial model specifications, 2021.

Exposure	SDI-adjusted	+ Super-region	Spatial error (k = 4)	Spatial error (k = 8)
Cataract-related vision loss				
Ambient particulate matter pollution	1.18 (1.07–1.31)*	1.07 (0.99–1.16)	1.14 (1.03–1.25)*	1.13 (1.04–1.24)*
Ambient ozone pollution	1.18 (1.08–1.28)*	1.08 (0.97–1.21)	1.07 (0.96–1.19)	1.06 (0.96–1.17)
High body-mass index	1.19 (1.05–1.35)*	1.06 (0.97–1.15)	1.10 (0.99–1.23)	1.12 (1.00–1.24)
Low bone mineral density	1.17 (1.03–1.33)*	1.00 (0.87–1.16)	1.12 (0.97–1.29)	1.02 (0.88–1.18)
High temperature	1.27 (1.18–1.37)*	1.21 (1.10–1.33)*	1.20 (1.10–1.32)*	1.22 (1.11–1.33)*
Glaucoma-related vision loss				
Ambient particulate matter pollution	1.27 (1.19–1.36)*	1.05 (0.99–1.12)	1.17 (1.09–1.25)*	1.13 (1.06–1.20)*
Ambient ozone pollution	1.14 (1.04–1.25)*	1.05 (0.97–1.14)	1.08 (0.99–1.17)	1.06 (0.98–1.14)
High body-mass index	1.22 (1.10–1.35)*	1.07 (1.01–1.13)	1.07 (0.98–1.16)	1.11 (1.03–1.19)
Low bone mineral density	1.23 (1.11–1.35)*	0.97 (0.87–1.08)	1.21 (1.09–1.34)*	1.13 (1.03–1.25)
High temperature	1.18 (1.10–1.27)*	1.09 (1.03–1.15)*	1.10 (1.03–1.18)	1.09 (1.02–1.16)

Values are country-level PRs (95% CIs) per 1-SD higher SEV. An asterisk (*) indicates outcome-specific BH q < 0.05 in the corresponding model specification. Each exposure was modeled separately with SDI adjustment. Full estimates and q values for all 26 exposures are presented in Supplementary Table S8.

PR, prevalence ratio; CI, confidence interval; SD, standard deviation; SEV, summary exposure value; SDI, Socio-demographic Index; BH, Benjamini–Hochberg; k, number of nearest neighbours.

### Model diagnostics

We examined Spearman correlations among the 26 candidate SEVs and SDI (Figure S2). For each primary association model, we also calculated the exposure variance inflation factor (VIF) relative to the SDI spline adjustment terms (Supplementary Table S10). As each model included only one SEV, these VIFs describe collinearity with the SDI adjustment rather than among all 26 SEVs in a single model. Model fit was summarized using in-sample R^2^, the share of between-country variation in log prevalence explained by the model, and root mean squared error (RMSE), the magnitude of the remaining error on the log scale. We used Ramsey’s regression equation specification error test (RESET) to assess possible departures from the specified model form and residual Moran’s I to examine remaining spatial dependence. Leverage and Cook’s distance were calculated across all 52 primary models to identify potentially influential countries. Further checks of influential countries and nonlinear exposure–response forms were restricted to the five exposures positive in both primary models (Supplementary Tables S10–S12).

### Random forests, SHAP, and validation

Supplementary random-forest models predicted log age-standardized prevalence. Forests including the 26 candidate SEVs and SDI used 500 trees, a minimum terminal-node size of five, and mtry = 9; SEV-only forests used mtry = 8. These settings were fixed rather than tuned. Each tree was fitted to a bootstrap sample drawn with replacement, with the same sample size as the training set.

Predictive performance was evaluated using five repetitions of five-fold cross-validation and leave-one-super-region-out validation. Within each validation split, models were fitted using training countries only, and corresponding comparisons used the same splits. The SDI-only comparator was a linear model with a three-degree-of-freedom natural spline of SDI, fitted within each training set. Root mean squared error (RMSE) and mean absolute error (MAE) were calculated on the log scale. Predictive R^2^ compared held-out prediction errors with errors from using the corresponding training-set outcome mean.

For forests fitted to all 204 countries with the 26 SEVs and SDI, marginal Shapley additive explanation (SHAP) values were estimated using a custom permutation-path implementation in R. A common background sample of 100 countries was used for both outcomes, with 100 paths evaluated for each explained country, one starting from each background country, and randomized predictor order within each path. Changes in predicted log prevalence along each path were attributed to the corresponding predictors. Mean absolute SHAP values summarized fitted predictive importance across countries. Because correlated predictors may share predictive information, these SHAP summaries do not isolate the independent contribution of any single exposure. Fixed random seeds were used for forest fitting, validation splits, background sampling, and permutation paths.

### Statistical software

All 204 countries and territories had complete data for the outcomes and predictors used in these analyses, so no imputation was required. Analyses were performed in R 4.6.0. Random forests used randomForest 4.7–1.2; data preparation and figures used dplyr 1.2.1, tidyr 1.3.2, and ggplot2 4.0.3. Regression models used stats and splines, with custom routines for robust covariance estimation, spatial likelihoods, and SHAP calculations.

## Results

### National burden and concordance in 2021

The prevalence of cataract-related vision loss was higher than that of glaucoma-related vision loss. Median national age-standardized prevalence in 2021 was 4,881.7 per 100,000 for cataract-related vision loss and 574.5 per 100,000 for glaucoma-related vision loss (Figures 1A,B). The corresponding 25th and 75th percentile cutpoints were 2,462.4 and 8,630.8 per 100,000 for cataract and 339.4 and 1,025.3 per 100,000 for glaucoma (Supplementary Table S2). The two vision-loss burdens varied geographically, with some similarities in their national distributions (Figures 1A,B). Countries in the highest quartile for cataract-related vision loss were found in Oceania, South and Southeast Asia, North Africa and the Middle East, and parts of sub-Saharan Africa. Fiji was among the Pacific island countries in this highest burden tier. For glaucoma, high-burden countries were concentrated in sub-Saharan Africa, with others in North Africa and the Middle East, including Egypt, Iran, and Saudi Arabia.

Using national age-standardized prevalence rates, we grouped countries and territories into three geographic concordance categories: Concordant, Cataract-dominant, and Glaucoma-dominant (Figures 1C,D; Supplementary Table S3). A total of 89 countries (43.6%) were Concordant. Within this group, 15 countries were in the highest quartile for both outcomes, mainly in North Africa, the Middle East, and parts of sub-Saharan Africa. These included Nigeria and Egypt. Countries in the lowest quartile for both outcomes were mainly in North America, Western and Central Europe, and Australasia (Figures 1C,D; Supplementary Table S3). The Cataract-dominant category included 52 countries (25.5%), with many in Oceania and South and Southeast Asia. India, Pakistan, China, and Bangladesh were among the countries in this category (Figures 1C,D; Supplementary Table S3). The Glaucoma-dominant category included 63 countries (30.9%). Most were in sub-Saharan Africa, with others in the Caribbean and parts of the Middle East. Examples included South Africa, Senegal, Iran, and Malawi (Figures 1C,D; Supplementary Table S3). The number of Concordant countries was 110 with tertiles and 72 with quintiles, compared with 89 under the quartile classification (Supplementary Table S1).

The 2021 national prevalence patterns also showed significant spatial structure. With four-nearest-neighbour weights, univariate Moran’s I was 0.620 for cataract-related vision loss and 0.797 for glaucoma-related vision loss (both permutation *p* = 0.0001). The directional bivariate statistics were 0.508 for cataract relative to neighbouring glaucoma burden and 0.442 for the reverse direction (both *p* = 0.0001). Results with eight-nearest-neighbour weights were similar (Supplementary Table S7).

### Temporal patterns, 2011–2021

The broad concordance pattern changed little between 2011 and 2021 (Figure 2). In 2011, 86 countries were Concordant, 53 Cataract-dominant, and 65 Glaucoma-dominant, compared with 89, 52, and 63, respectively, in 2021. Across the 11 annual classifications, 191 of 204 countries (93.6%) remained in the same concordance category, and 12 were in the highest quartile for both outcomes every year (Supplementary Table S3). Figure S1 shows annual median national prevalence within each GBD super-region, while Supplementary Table S4 reports the classification using fixed 2021 cutpoints.

### Primary exposure associations

Across the 26 candidate exposures, five had positive coefficients with outcome-specific BH q < 0.05 for both cataract- and glaucoma-related vision loss in the primary SDI-adjusted models: ambient particulate matter pollution, ambient ozone pollution, high body-mass index, low bone mineral density, and high temperature (Figure 3; Supplementary Table S6). In the cataract models, the prevalence ratios were 1.269 (95% CI 1.178–1.367) for high temperature and 1.183 (1.068–1.310) for ambient particulate matter pollution. For glaucoma, ambient particulate matter pollution showed a PR of 1.270 (1.185–1.360), while low bone mineral density showed a PR of 1.226 (1.109–1.354). Each PR represents the country-level association between a one-SD higher SEV and age-standardized prevalence.

Among exposures meeting the positive criterion in one primary model only, secondhand smoke (PR 1.276, 95% CI 1.176–1.384), high fasting plasma glucose (1.230, 1.133–1.334), low physical activity, and low temperature were identified in the cataract models. Lead exposure met the corresponding criterion for glaucoma (PR 1.137, 95% CI 1.045–1.238) but not cataract. Figure 3 and Supplementary Table S6 show all 52 primary estimates, including inverse and nonsignificant associations.

### Regional and spatial sensitivity

The primary associations showed substantial sensitivity to regional and spatial adjustment (Figure 4; Table 1). Of the five exposures positive for both outcomes in the primary models, only high temperature continued to meet the same two-outcome criterion after adjustment for GBD super-region. Ambient particulate matter pollution was the only exposure to meet it for both outcomes in both the four- and eight-nearest-neighbour spatial-error models. No exposure remained positive for both outcomes with BH q < 0.05 in all four model specifications. Complete results for all 26 exposures are provided in Supplementary Table S8. Region-omission analyses showed that some estimates changed when individual super-regions were excluded (Supplementary Table S9).

### Diagnostics and influence

Correlations among the 26 candidate SEVs and SDI are shown in Supplementary Figure S2. The highest exposure VIF was 6.30 for household air pollution from solid fuels (Supplementary Table S10). Across the 52 primary models, R^2^ ranged from 0.411 to 0.571 for cataract and from 0.525 to 0.704 for glaucoma; log-scale RMSE ranged from 0.468 to 0.549 and from 0.363 to 0.460, respectively. RESET *p* values were <0.05 in 16 models, and residual Moran’s I showed remaining spatial dependence. Additional checks of model form and influential countries for the five exposures positive in both primary models are reported in Supplementary Tables S11, S12.

### Supplementary prediction analyses

In repeated five-fold cross-validation, the random forest including the 26 SEVs and SDI achieved R^2^ values of 0.687 for cataract and 0.794 for glaucoma. When one GBD super-region at a time was left out for validation, R^2^ fell to 0.347 and 0.369, respectively. These values were similar to those from the SDI-only models: 0.337 for cataract and 0.373 for glaucoma. SHAP importance and full validation results are reported in Supplementary Tables S13, S14.

## Discussion

This country-level analysis found geographic concordance in cataract- and glaucoma-related vision-loss burdens across 204 countries and territories. Among adults aged 55 years and older, 89 of 204 countries and territories were Concordant, and 15 were in the highest quartile for both outcomes, mainly in North Africa, the Middle East, and parts of sub-Saharan Africa. In the primary SDI-adjusted models, five exposures had positive associations with both outcomes, although statistical support varied after regional and spatial adjustment.

Previous studies have reported that cataract and glaucoma often occur together in older adults (7). These studies examined coexisting eye disease in specific populations (6, 7), whereas our analysis addresses a different question: how the national burdens of cataract- and glaucoma-related vision loss align geographically. Because countries rather than individuals were the units of analysis, geographic concordance and exposure associations should not be interpreted as individual-level co-occurrence or risk relationships. The number of countries in each category varied with the number of burden tiers. Across 2011–2021, 191 of 204 countries remained in the same concordance category, and 12 remained in the highest quartile for both outcomes every year. However, stable category assignments do not necessarily imply stable prevalence rates or country rankings for either outcome. The Moran analyses also showed spatial clustering of each outcome and a positive spatial association between cataract and glaucoma burdens in neighbouring countries. Geographic variation in age-related cataract prevalence has also been reported previously (18). These spatial features may reflect differences in socioeconomic development and the distribution of healthcare resources. Undiagnosed age-related eye disease has been documented in older adults in some settings (19), suggesting that differences in case detection may also affect observed burden estimates. Both conditions place demands on eye-care services, from cataract surgery to glaucoma assessment and long-term follow-up (20–22). These geographic patterns may help inform eye-care planning in settings where both conditions contribute substantially to vision-loss burden.

After adjustment for SDI, ambient particulate matter pollution, ambient ozone pollution, high body-mass index, low bone mineral density, and high temperature showed positive associations with both vision-loss outcomes in the primary models. However, these findings were sensitive to geographic adjustment. Only high temperature retained this pattern after adjustment for GBD super-region, whereas ambient particulate matter pollution retained it in both spatial-error models. No exposure showed the same statistically supported positive association with both outcomes across all four model specifications. Region-omission analyses also showed that some estimates changed when individual super-regions were excluded. Because exposures were modeled separately, these estimates represent SDI-adjusted country-level associations rather than mutually adjusted exposure effects.

Previous human studies provide context for several of these findings. Long-term exposure to ambient PM2.5 has been associated with age-related cataract in cohort studies and with incident glaucoma in the UK Biobank (23, 24). Higher ambient temperature has also been associated with cataract, while heatwaves have been associated with glaucoma (25, 26). Evidence for the remaining common-positive exposures is less consistent across the two diseases. A meta-analysis of prospective cohorts found higher BMI to be associated with age-related cataract, whereas multicohort glaucoma data suggest a more complex relationship with BMI (27, 28). Recent prospective cohort data have also linked osteoporosis or low bone mineral density with a higher risk of age-related cataract (29). Taken together with the changes observed after regional and spatial adjustment, these studies support cautious interpretation of the country-level exposure findings rather than treating them as established shared risk factors. Interpretation of the glaucoma findings is further limited by the pooled GBD outcome, which combines subtypes with different geographic distributions and associated factors.

Several exposures met the positive criterion for only one outcome. For cataract, these included secondhand smoke, high fasting plasma glucose, low physical activity, and low temperature. Among these, higher fasting glucose has also been associated with cataract incidence and progression in the Blue Mountains Eye Study (30). Lead exposure met the positive criterion only for glaucoma in our primary models, although previous cohort studies have examined cumulative lead exposure in relation to both age-related cataract and incident primary open-angle glaucoma (31, 32).

The random forests performed better in repeated five-fold cross-validation than when entire super-regions were held out. Under regional validation, the forests including SEVs and SDI performed similarly to the SDI-only spline models. This suggests that predictive patterns learned across countries may not generalize well to new geographic regions. SHAP rankings describe contributions to fitted predictions rather than independent exposure associations. For public health practice, the geographic patterns may help inform the coordination of cataract care and glaucoma assessment, while the ecological exposure findings provide hypotheses for further epidemiological research.

## Strengths and limitations

A major strength of this study is its global assessment of geographic concordance in cataract- and glaucoma-related vision-loss burdens. Regional and spatial sensitivity analyses, together with cross-region prediction validation, allowed us to examine how the findings varied across geographic settings. Several limitations should also be noted. Because we used country-level cross-sectional aggregated data, the observed associations cannot establish causality and may not reflect individual-level risk relationships. Although the exposure models adjusted for SDI, differences in healthcare access, screening, case detection, and other country-level conditions may still confound the associations and contribute to under-ascertainment in lower-resource settings. The vision-loss outcomes and SEVs are also model-derived GBD estimates based on data that vary in quality and availability across countries. The reported confidence intervals reflect uncertainty in the fitted models but do not incorporate the full uncertainty of the underlying GBD estimates. Differences in diagnostic criteria, screening coverage, and reporting systems may further affect comparisons across countries. The GBD glaucoma-related vision-loss outcome in our analytic dataset does not distinguish between open-angle and angle-closure glaucoma, which differ in their geographic distributions and associated factors; the pooled associations may therefore partly reflect differences in subtype composition. Cross-sectional exposure metrics may not capture long-term cumulative exposure or lag effects. The candidate-exposure panel was informed by published studies but was not prospectively prespecified, so the exposure findings remain exploratory. Correlations among the SEVs and with SDI may also complicate the interpretation of individual exposure estimates and SHAP importance. Finally, HC3 robust covariance estimates do not account for spatial dependence between countries, and some primary models showed residual spatial clustering or evidence of model-form inadequacy.

## Conclusion

Across 204 countries and territories, cataract- and glaucoma-related vision-loss burdens showed geographic concordance among adults aged 55 years and older. Five candidate exposures had positive associations with both outcomes in the primary SDI-adjusted models, but the results were sensitive to regional and spatial adjustment. These national patterns may inform eye-care planning, while the ecological exposure associations warrant further investigation at the individual level.

## Data Availability

The original contributions presented in the study are included in the article/Supplementary material, further inquiries can be directed to the corresponding author.

## Supplementary material

SUPPLEMENTARY FIGURE S1 Regional trajectories of standardized vision-loss prevalence, 2011–2021. **(A)** Cataract; **(B)** glaucoma. Lines and annual symbols represent the median national rate within each GBD super-region, using fixed 2021 Global 55+ reference weights. These are unweighted country medians, not population-weighted regional prevalence estimates. Regions are labeled directly, with leader lines connecting the labels to their 2021 values. Both vertical axes begin at zero; the two outcome panels use different vertical scales.

SUPPLEMENTARY FIGURE S2 Correlations among the 26 candidate SEVs and SDI, 2021. The lower triangle shows all 351 unique country-level Spearman correlations across 204 countries. Hierarchical ordering uses one minus the absolute correlation and is identical for rows and columns. Column numbers correspond to the numbered row labels. Coral and blue indicate positive and negative correlations, respectively, with a common color scale from −1 to 1. Diagonal cells represent self-correlations of 1 and are shown in grey, outside the color scale. Short labels correspond to full exposure names in Supplementary Table S6.
